# Immunogenicity of the BNT162b2 mRNA COVID-19 vaccine in older residents of a long-term care facility: relation with age, frailty and prior infection status

**DOI:** 10.1007/s10522-021-09944-9

**Published:** 2021-12-19

**Authors:** Piotr Seiffert, Adam Konka, Janusz Kasperczyk, Jacek Kawa, Mateusz Lejawa, Barbara Maślanka-Seiffert, Joanna Zembala-John, Monika Bugdol, Małgorzata Romanik, Rafał Bułdak, Czesław Marcisz, Jarosław Derejczyk, Dorota Religa

**Affiliations:** 1 Department of Gerontology and Geriatric Nursing, School of Health Sciences, Medical University of Silesia, Katowice, Poland; 2 Silesian Park of Medical Technology Kardio-Med Silesia, Zabrze, Poland; 3 Chair and Department of Medicine and Environmental Epidemiology, Faculty of Medical Sciences in Zabrze, Medical University of Silesia, Katowice, Poland; 4 Faculty of Biomedical Engineering, Silesian University of Technology, Zabrze, Poland; 5 Department of Pharmacology, Faculty of Medical Sciences in Zabrze, Medical University of Silesia, Katowice, Poland; 6 Geriatric Ward, The Municipal Hospital, Chorzow, Poland; 7 Silesian Center for Heart Diseases, Zabrze, Poland; 8 Department of Medical Microbiology, School of Medicine in Katowice, Medical University of Silesia, Katowice, Poland; 9 Department of Clinical Biochemistry and Laboratory Diagnostics, Institute of Medical Sciences, University of Opole, Opole, Poland; 10 Long-Term Care Facility “proAltum”, Myslowice, Poland; 11 Division of Clinical Geriatrics, Department of Neurobiology, Care Sciences and Society, Karolinska Institutet, Huddinge, Sweden; 12 Theme Aging, Karolinska University Hospital, Huddinge, Sweden; 13 Rheumatology Ward, Murcki Hospital, Katowice, Poland

**Keywords:** COVID-19, COVID-19 vaccines, Frailty, SARS-CoV-2, Long-term care, Older adults

## Abstract

Clinical and biological assessment of the COVID-19 vaccine efficacy in the frail population is of crucial importance. The study focuses on measuring the levels of anti-SARS-CoV-2 IgG antibodies before and after BNT162b2 mRNA COVID-19 vaccination among long-term care facility (LTCF) elderly residents. We conducted a prospective, single-center, observational study among LTCF residents. The study protocol was based on three blood sample acquisitions: first taken at baseline—5 days before the first dose of the vaccine, second—20 days after the first dose, and third—12 days after the second shot of the vaccine. The comparison was made for two cohorts: patients with and without prior COVID-19 infection. The data was collected from January to March 2021. A total number of 78 LTCF residents (55 women and 23 men) aged 62–104, 85.72 ± 7.59 years (mean ± SD), were enrolled in the study. All study participants were investigated for the presence of SARS-CoV-2 anti-spike (S) protein IgG, using a chemiluminescent immunoassay. Frailty was assessed with the Clinical Frailty Scale. Among elderly COVID-19 survivors in LTCF, a single dose of vaccine significantly increased anti-SARS-CoV-2 IgG antibody levels. IgG concentration after a single and double dose was comparable, which may suggest that elderly COVID-19 survivors do not require a second dose of vaccine. For residents without a previous history of COVID-19, two doses are needed to achieve an effective serological response. The level of anti-SARS-CoV-2 IgG antibodies after vaccination with BNT162b2 mRNA COVID-19 did not correlate with the frailty and age of the studied individuals.

**Supplementary Information:**

The online version contains supplementary material available at 10.1007/s10522-021-09944-9.

## Introduction

The COVID-19 pandemic revealed many gaps in our current knowledge about mechanisms underpinning disease susceptibility and maladaptation related to the aging process. As older age is one of the main risk factors for SARS-CoV-2 mortality, biogerontology should become an integral part of global public health priorities (Farrelly 2021). The effect of age on mortality from COVID-19 has been observed with the relevant thresholds on age > 50 years and the highest mortality rate in patients aged ≥ 80 (Bonanad et al. 2020). What is more, the frailty level, frequently high in older adults, has been recognized as a useful predictor of mortality coronavirus risk in geriatric patients (Apea et al. 2020; Bielza et al. 2021; Aliberti et al. 2021). As the long-term care facility (LTCF) residents represent an older and more frail population, the clinical importance of the above-mentioned is easily observed—LTCF residents account for 30–40% of all deaths due to COVID-19 (Rolland et al. 2021). However, those patients suffer not only from COVID-19 itself. Isolation and long-term loneliness, forced by implemented national/local sanctions, constitute an additional burden in this group, affecting both physical and mental health (Van der Roest et al. 2020) but also frailty development (Davies et al. 2021). Having the above mentioned facts in mind, most countries considered vaccinating LTCF residents as a priority.

COVID-19 mRNA vaccines provide effective protection against infection with SARS-CoV-2 virus (Polack et al. 2020; Baden et al. 2021). However, LTCF residents have not been represented in most vaccine development research. What is more, data about vaccination reactogenicity and immunogenicity in the oldest adults is still limited. The described situation generates differences both at the level of national policies and vaccination regimens of individuals. Careful consideration regarding a second dose of vaccine is given to older residents living with frailty who experienced severe adverse effects after the vaccine’s first dose. Also, little is known about profit balance in patients with a very short life expectancy. In patients with previous COVID-19 infection, one dose of mRNA vaccine induces rapid immune responses similar to or exceeded titers found in seronegative participants who received full vaccination, but whether a single dose of mRNA vaccine provides an effective protection against infection still requires investigation (Krammer et al. 2021).

The effect of vaccination can be observed on many levels. Vaccines efficacy against COVID-19 and reduction in frequency of severe disease are one of the most common endpoints of vaccines development research (Polack et al. 2020). On the society level, epidemiological research reports both numbers of avoided deaths and numbers of years of life lost as the effect measure of the vaccination or reductions in costs resulting from the decreased need for treatment (Bloom et al. 2021). Measurement of the immune response is one of the main ways of exploration on the biological level (Knezevic et al 2021). Immunological parameters describe the humoral or the cell-mediated immune response. As the immune system is a complex network of specialised cells and tissues that communicate through lots of agents, many techniques can be used to assess the response, e.g. flow and mass cytometry (for immune cell phenotyping), protein and peptide microarrays (for antibody profiling), microarray and RNA sequencing (for immune cell gene expression), metabolomics (for immune cell metabolic state), luminex and mesoscale (for cytokine determination) and next-generation sequencing (for IgG and T-cell receptor repertoire analysis) (Furman and Davies 2015). In spite of all, the focus is usually on determination of antibody levels, as parameters other than those that measure the humoral immune response have not played a pivotal or major role in vaccine licensure (WHO Technical Report 2017). The assessment of the efficacy of the antibodies response to vaccination can be performed not only by measuring the antibody titers but also on assessing their quality e.g., their avidity, specificity, or neutralizing capacity.

The mass and simultaneous vaccination will undoubtedly reveal differences in vaccination responses of individuals worldwide. It would be of special interest to identify variables other than age in this heterogeneous group. As frailty affects older adults’ responses to vaccines (Andrew and McElhaney 2021) careful consideration should be given in assessing its role in COVID-19. Understanding the expected antibody response in LTCF residents may facilitate selecting the optimal vaccination protocol for the frailest elderly. It may also enable estimating the risk of potential adverse effects and new infection onset.

This study aimed to measure the levels of anti-SARS-CoV-2 IgG antibodies and conduct their timeline observation after COVID-19 vaccination among LTCF residents and compare the results between two cohorts: patients with and without prior COVID-19 infection. The impact of age and frailty on IgG concentration was also analyzed.

## Methods

We conducted a prospective, single-center, observational study to analyze the seroprevalence of anti-SARS-CoV-2 IgG antibodies among elderly residents of an LTCF. The study got the approval of the Ethics Committee and was conducted in accordance with the declaration of Helsinki. The data was collected between 01.2021 and 03.2021. In vaccinated patients, the response to mRNA BNT162b2 vaccine (Pfizer-BioNTech) was examined.

The inclusion criteria were: written informed consent in person or by a legal guardian and a LTCF resident status. The exclusion criterion was a lack of consent. Frailty was assessed with the Clinical Frailty Scale [CFS] by two geriatric consultants at the beginning of the study (Rockwood and Theou 2020). CFS is an effective measure of frailty and provides predictive information similar to that of other established tools about death or the need for an institution. CFS is an easy to use, not time-consuming inclusive 9-point scale that is developed to to determine overall level of fitness or frailty (from “very fit” to “terminally ill”). Data about adverse vaccine reactions were collected from the LTCF physicians' and nurses' documentation and from the examination of the residents. In the analyzed LTCF, the outbreak of COVID-19 infection occurred in October and November 2020. All data were anonymized on an ongoing basis.

All the included residents were investigated for the presence of SARS-CoV-2 anti-spike (S) protein IgG, using a chemiluminescent immunoassay. Measurement of serum levels of IgG antibodies was performed using Access SARS-CoV-2 IgG II assay (Beckman Coulter, USA) on the Access 2 Immunoassay System (Beckman Coulter, USA).

For all participants, we quantified circulating levels of IgG antibodies at baseline prior to vaccination, after the first dose of the vaccine, and after the second dose of the vaccine. The study protocol was based on three blood samples acquisitions:

1. 5 days before the first dose of the vaccine in LTCF (previously available data),
2. 20 days after the first dose of the vaccine in LTCF,
3. 12 days after the second dose of the vaccine in LTCF.

**Fig. 1:**
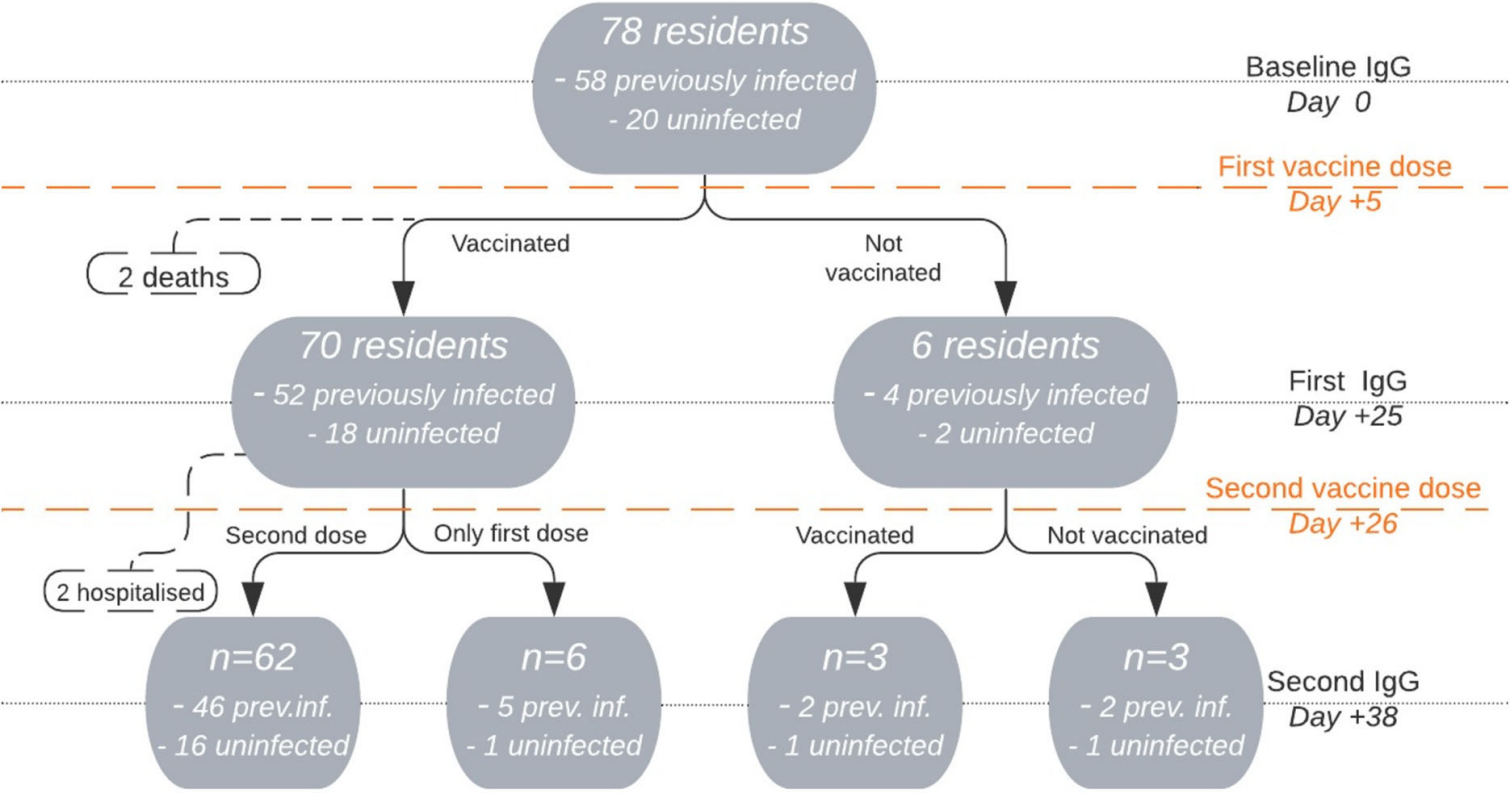
Study flowchart

The study flowchart is presented in Fig. 1. In the examined LTCF two ‘vaccination days’ were organized. The vaccination and blood samples collection were performed on the same day for all patients.

We compared antibody levels between these with and without prior SARS-CoV-2 infection. Previous COVID-19 diagnosis was confirmed by positive detection of antibodies against the SARS-CoV-2 at baseline or history of a previous positive diagnostic test. We analyzed data at baseline and following dose 1 and dose 2.

**Fig. 2:**
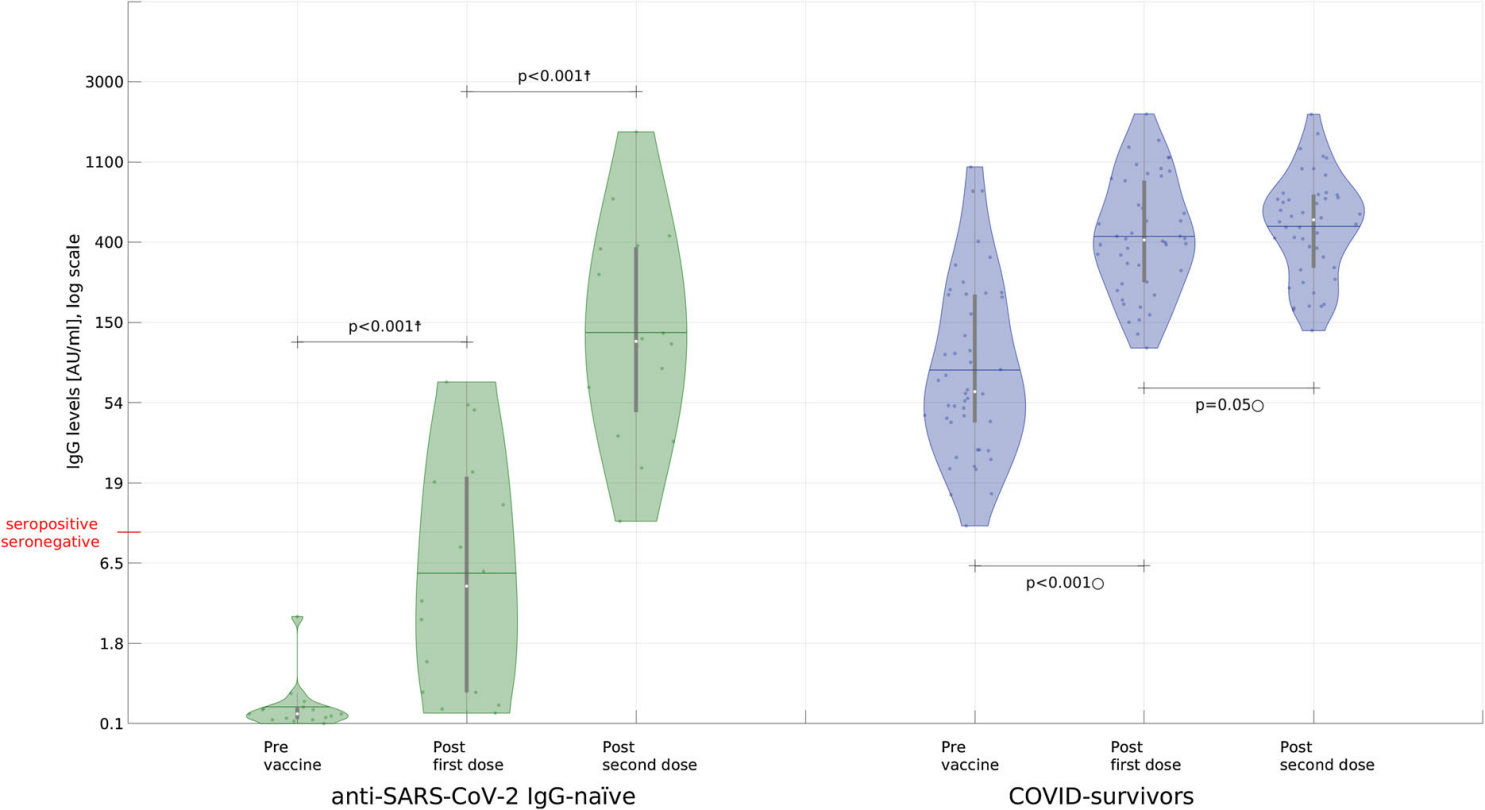
Serological response to the first and the second dose of the BNT162b2 mRNA COVID-19 vaccine in individuals with and without laboratory-confirmed previous SARS-CoV-2 infection (y-axis is log-scaled ln(0.9 + ○) with raw values; whiskers mark mean and location of the 25/75 percentile/IQR) only a group with two doses of vaccine is presented, n = 62. After the first dose, in a group without previous SARS-CoV-2 infection 10 patients were still seronegative. The † symbol marks large effect in the Friedman test whereas corresponding p-values were obtained for the post hoc pairwise Wilcoxon test with Holm–Bonferroni adjustment; the ○ symbol marks large effect in ANOVA test whereas corresponding p-values were obtained for the post hoc pairwise *t* test with Holm–Bonferroni adjustment

**Fig. 3:**
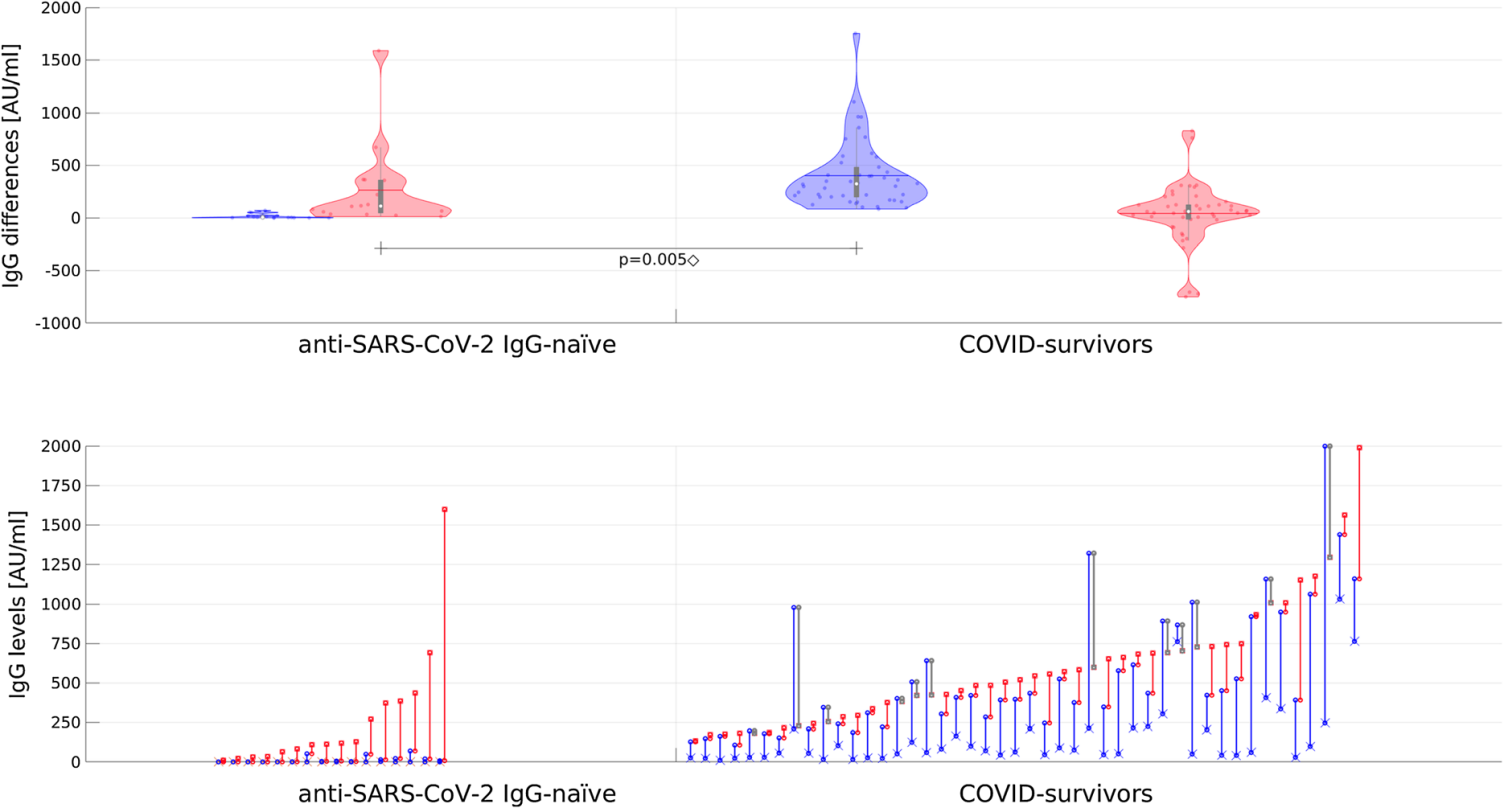
The effects of the first (blue) and second (red) BNT162b2 mRNA COVID-19 vaccine dose as a difference between anti-S titres noted during consecutive examinations (whiskers mark mean and IQR; y-axis is linear; only a group with two doses of vaccine is presented; n = 62). Lower chart shows the matching anti-S titers, sorted by the last measured level (blue and red denote positive change after, respectively, first and second dose; cases with the level lower after the second dose are colored gray). For clarity, the initial level is additionally marked with x while the final level (observed after second dose) with square. The ◇ symbol denotes a large effect in Kolmogorov–Smirnov test (p < 0.001) and Mann–Whitney test (p = 0.005)

For the study, long-transformation of non-normally distributed data was used. Antibodies levels within seronegative and seropositive groups were compared using the ANOVA multiple comparison test (where applicable) or the Friedman test. Post hoc, the pairwise Wilcoxon signed-rank test or the pairwise *t* test with Holm–Bonferroni adjustment were applied. Growth distributions (raw differences) were subjected to the Ansari–Bradley and Kolmogorov–Smirnov tests. Independent samples, using *t*-test, Welch test, or Mann–Whitney *U* test/Wilcoxon rank-sum test were compared. Based on individual samples, the most powerful available tests were always applied. Statistical analysis was performed using R 4.0.4. Figures 2, 3 were prepared in Matlab 2020b. p-value of 0.05 (p = 0.05) was considered statistically significant.

## Results

### Prior infection status

Total number of 78 LTCF residents (55 women and 23 men) aged 62–104, 85.72 ± 7.59 years (mean ± SD), all white, who met the inclusion criteria were enrolled into the study. The characteristics of the study sample, CFS score and IgG measures are presented in Table 1.

**Table 1:** General characteristics, Clinical Frailty Scale scores and the serum level of anti-SARS-CoV-2 IgG antibodies in serum in the studied long-term care facility residents before and after vaccination, taking into account SARS-CoV-2 infection

Parameter	Investigated patients							
	Pre-vaccine		Post-vaccine First dose		Post-vaccine Second dose		Only first dose regimen	
	Uninfected (n = 20)	Previously infected (n = 58)	Uninfected (n = 18)	Previously infected (n = 52)	Uninfected (n = 16)	Previously infected (n = 46)	Uninfected (n = 1)	Previously infected (n = 5)
Age (years; mean ± SD)	85.55 ± 7.16	85.78 ± 7.79	84.61 ± 6.01	85.56 ± 8.01	84.31 ± 6.29	85.09 ± 8.25	85	88.80 ± 5.40
Sex (male/female)	10/10	13/45	9/9	12/40	8/8	11/35	0/1	1/4
sCFS (median/IQR/range)	5/4/1–7	6/2/3–8	5/4/1–7	6/2/3–8	5/3.5/1–7	6/2/3–8	5//5	6/2.2/4–7
Serum level of anti-SARS-CoV-2 IgG (AU/ml) (median/IQR/range)	0.23/0.17/0.10–4.69	73.56/ 168.55/ 10.86–1372.23	4.76/21.59/ 0.24–232.96	433.58/670.40/ 107.02–3956.48	116.50/328.00/ 11.56–1597.56	531.59/433.64/ 133.25–1989.15	0.60	988.15/468.90/ 354.17–1114.69

*CFS* Clinical Frailty Scale, *IQR* interquartile range

62 residents (79.48% of all residents) were vaccinated with two doses of mRNA vaccine. The characteristics of this group are presented in Table 2. In this cohort, none of the seronegative individuals had been previously tested positive for COVID-19, while among seropositive individuals 29 (63%) in 46 had a history of previously positive PCR result.

**Table 2:** Serum level of anti-SARS-CoV-2 IgG in subjects vaccinated with BNT162b2 mRNA COVID-19 vaccine

Serum level of anti-SARS-CoV-2 IgG (AU/ml)						
	Pre-vaccine		Post first dose		Post second dose	
	Uninfected (n = 16)	Previously infected (n = 46)	Uninfected (n = 16)	Previously infected (n = 46)	Uninfected (n = 16)	Previously infected (n = 46)
Mean ± SD	0.42 ± 0.68	151.10 ± 211.46	15.68 ± 22.02	552.39 ± 409.67	279.47 ± 399.82	594.57 ± 385.61
Median/IQR/range	0.23/0.16/0.10–2.91	61.72/167.06/10.86–1030.52	4.76/20.25/0.24–69.79	413.44/621.84/107.02–998.33	116.50/328.00/11.56–1597.56	531.59/433.64/133.25–1989.15

*SD* standard deviation, *IQR* interquartile range

#### Seronegative Subgroup

Among seronegative individuals one dose of vaccine significantly increased IgG levels (post-hoc pairwise Wilcoxon test p < 0.001; ratio of medians 20.69, ratio of means 37.7). Still, in this cohort 10 in 16 residents were anti-SARS-CoV-2 IgG naive after the first dose. After the second dose, the IgG level growth was significantly higher than after the first one (pairwise Wilcoxon test with Holm–Bonferroni adjustment p < 0.001, large effect; ratio of medians 24.47, ratio of means 17.8) (Figs. 2, 3).

#### Seropositive Subgroup

Among those with a previous SARS-CoV-2 infection, one dose of vaccine significantly increased anti-SARS-CoV-2 IgG from peak pre-vaccine levels (post hoc pairwise *t*-test with Holm–Bonferroni adjustment p < 0.001; the ratio of medians 6.69, the ratio of means 3.65) (Figs. 2, 3). IgG response after the second dose was significantly lower than after the first dose (pairwise Wilcoxon with Holm–Bonferroni adjustment, p < 0.001, large effect; the ratio of medians 1.28, the ratio of means 1.07) and in 12 participants (26.1%) lowering of IgG levels was observed.

#### Response After the First Dose

Among seronegative individuals, anti-S titers after one vaccine dose were lower to peak anti-S titers in individuals with a previous natural infection (about 3 months after COVID-19 outbreak in the LTCF) who had not yet been vaccinated (unpaired *t*-test p < 0.001) but comparable after the second dose (unpaired *t*-test p > 0.05) (Figs. 2, 3).

#### Response After the Second Dose

Among seronegative individuals after the second dose IgG levels were lower than after one dose in COVID-19 survivors group (Welch’s *t*-test p < 0.005) (Figs. 2, 3).

### Frailty

The group was frail (median CFS score 6, interquartile range (IQR) = 2) without a significant difference between COVID-19 survivors and anti-SARS-CoV-2 IgG naive patients (Wilcoxon rank-sum test p = 0.08). However, a significant CFS score difference (Wilcoxon rank-sum test p < 0.05) was observed in a group qualified for two doses of vaccine (median = 6, IQR = 2 vs median = 5 IQR = 3.5 between COVID-19 survivors and anti-SARS-CoV-2 IgG naive patients, respectively).

No statistically significant correlation between CFS score and IgG levels or IgG growth responses was observed.

### Age

Both the Pearson and Spearman correlation were calculated and their statistical significance were verified using the significance test for the correlation coefficient. No significant correlation between anti-SARS-CoV-2 IgG levels and IgG growth responses and age in any of the cohorts was confirmed.

### Sex

No significant differences in anti-SARS-CoV-2 IgG levels and IgG growth responses between male and female were observed in COVID-19 naive individuals. In COVID-19 survivors group no significant differences were observed between male and females IgG levels. However anti-SARS-CoV-2 IgG growth after the second dose was bigger in males after the second dose than in females (p < 0.05).

### Vaccination Protocol Safety and Efficacy

Six patients were not vaccinated with the first dose: three because of lack of consent, another three were disqualified due to contraindications (in one case due to altered INR, another two because of acute infection).

After the first dose, six patients were not qualified by the physicians for the second dose: three because of acute infection, three (all survivors) due to poor tolerance of the first dose.

After the first dose fever and general weakness were detected in 9 patients (all COVID-19 survivors) with a maximum duration of two days. Also, pain and injection-site redness was observed in two patients.

After the second dose fever was noted in three patients. Pain and redness were reported by one participant. Two patients were hospitalized: one with the episode of significant hyperglycemia, second—due to recurrent significant anemia. During the study follow-up period (50 days after the first dose), two residents—both COVID-19 survivors died. Deaths occurred about a week and a month after the first dose without a proven relationship to the vaccine.

There was no new COVID-19 infection in either group.

## Discussion

Data about the durability of IgG response to natural infection in the LTCF population is limited. Oved et al. 2020 reported that 5% of COVID-19 patients do not develop IgG antibodies and that most serological assays identify antibodies in 85–90% of infected individuals (Oved et al 2020). Some studies suggest that IgG response decreases over time but is still present in a vast majority of LTCF residents up to 6 months after the infection (Ruopp et al. 2021). After mRNA vaccination serum neutralizing antibodies in healthy adults can be detected in all the subjects after 119 days (Widge et al 2021). No correlates of protection against COVID-19 infection were defined so far. Also, little is known about IgG thresholds required for vaccine efficacy (Ewer et al. 2021).

The most important finding of our study was that the first dose of vaccine in COVID-19 seropositive residents of LTCF acted as a booster dose, as previously reported in younger groups (Bradley et al 2021; Ebinger et al 2021; Manisty et al 2021). It is also comprehensive with the result in LTCF population reported by Blain et al. (2021). The IgG growth after the second dose was significantly lower, and decreases in antibodies level were also observed in approximately 25% of the seropositive residents. It is noteworthy that the highest final IgG levels were observed in COVID-19 survivors that were vaccinated only with the first dose. However, the small size of the group (n = 5) and its specification (i.a. people with poor tolerance of the first dose) limits the statistical importance of the observation. Rapid and robust IgG response may suggest that S protein, used in the BNT162B2 vaccine, is highly immunogenic. Effective immunogenicity was observed in LTCF residents also in other COVID-19 vaccines (Nace et al. 2021). Another argument for a vaccination of COVID-19 recovered individuals are observations that a single dose of mRNA vaccine boosted neutralising titers also against other variants of the virus (Stamatatos et al 2021). The immune system undergoes aging-related changes (Li et al. 2021), which continuously progress to a state of immunosenescence. The aging immune system loses the ability to protect against infections and cancer and fails to support appropriate wound healing. The immunosenescence observed in the elderly had been previously considered as one of the possible causes of hypothetically unsuccessful humoral immune response to vaccines (Turner and Mabbott 2017). Conversely, inflammatory responses mediated by the innate immune system gain in intensity and duration, rendering older individuals susceptible to tissue-damaging immunity and inflammatory disease (Weyand and Goronzy 2016). Alterations resulting from aging in the innate and signaling pathways have a crucial role in what is called “inflammaging” or the chronic basal production of pro-inflammatory cytokines like interleukin-6 (IL-6), tumor necrosis factor-α (TNF-α), and IFNs. Inflammaging can predict frailty and mortality in those aged 65 years and older when compared with counterparts without chronic inflammation (Franceschi et al. 2017). The initiation and control of the immune response depends on the function of dendritic cells (DCs). Their role in bridging innate and adaptive immunity in aging, however, is poorly understood in humans. In response to TLR 3, TLR 7/8, and TLR 9 ligand stimulation, primary mDCs (cDC1) and pDCs had lower levels of IL-6, IL-12, and TNF-α and surface expression of the TLRs but higher basal levels when compared with young donors (Connors et al. 2021). The age-related reductions in expression and activation of TLR, RLR, and inflammasome pathways have likely contributed to defects in response to viral infections resulting in age-linked susceptibility to relevant viruses like SARS-CoV-2. In contrast, our study revealed that a two-dose regimen effectively stimulated antibody response in all seronegative LTCF residents: after the second dose, IgG were detectable in all the subjects. However, one dose of vaccine in anti-SARS-CoV-2 IgG naive residents did not exceed the limit value in most subjects. Our study showed no correlation between anti-SARS-CoV-2 IgG levels and age. This may be explained by the fact that our residents were homogeneously older than in other studies. On the society level, mathematical analysis made by Goldstein et al. reveal that for COVID-19 vaccinating first the oldest old saves the most lives and also the most life left (Goldstein et al. 2021). We didn’t find differences in antibody levels according to sex, which differs from previous studies (Levin et al. 2021) that revealed higher IgG levels in women than in men also in older groups. On the other hand Dan et al. (2021) observed that males had higher spike IgG levels after COVID-19 infection.

Frailty is a state of an increased vulnerability to stressors caused by decline in physiological reserve in multiple physiological systems (Ghachem et al 2021; Seiffert et al 2017). The concept of frailty assessment is not new, yet in a last year, in time of the pandemic, it has caught clinicians' and researchers' particular attention worldwide. One of the main limitations of the frailty concept is the lack of a unified diagnostic standard. The two principal existing models of frailty are the phenotype model (Fried et al. 2001) and the cumulative deficit model (Rockwood et al 2005). Based on the second one, the Clinical Frailty Scale that we used in the study, is a 9-point scale (the higher scores mean greater risk) that is being used both in prognosis and to set care goals (Rockwood and Theou 2020). Frailty prevalence is much higher in the elderly, especially among LTCF residents (Kanwar et al 2013). Our study revealed no significant difference in baseline CFS score between COVID-19 survivors and anti-SARS-CoV-2 IgG naive patients in a group of all 78 residents. There was, however, a statistical difference in CFS level between anti-SAR-CoV-2 IgG naive patients and COVID-19 survivors (median CFS 5 vs CFS 6, respectively). However, it has been observed that it was easier to isolate patients with lower CFS scores. Besides, their better functional status allowed them to minimize their contact with other residents and staff during COVID-19 outbreak. Previous studies indicate that COVID-19 outbreak and isolation can accelerate the frailty progression of institutionalized elderly by around 20% (Greco et al 2021). Frailty affects older adults’ responses to vaccines (Andrew and McElhaney 2021). In contrast, our study did not reveal significant dependence of frailty in the IgG response to COVID-19 mRNA vaccine. Our observation is complementary with the similar study by Salmeron Rios et al. (2021), which suggests that the BNT162b2 mRNA COVID‐19 vaccine in older adults produces immunogenicity, independently of the frailty and disability profiles.

The main limitation of our study was a relatively small group of residents. This affected especially comparative analyzes of the subgroups, for example, the effect of gender. Moreover, the previously infected group was almost three times larger than IgG naive one. Furthermore, we only focused on IgG measurement while immunization can also be generated involving other types of immune memory (i.e., memory B cells, memory CD8+T cells, memory CD4+T cells). There are studies suggesting that circulating antibody titers are not predictive of T cell memory; therefore simple serological tests for SARS-CoV-2 antibodies do not reflect the richness and durability of immune memory to SARS-CoV-2 (Dan et al 2021).

Comparative assessment of the dynamics of changes in IgG concentration requires longer observation over time. Additional studies are needed to validate our findings, allowing for the personalization of vaccination regimens in frail, older LTCF residents.

## Conclusions and Implications

The effectiveness of the BNT162b2 mRNA COVID-19 vaccine as measured by the serum level of anti-SARS-CoV-2 IgG antibodies varied widely in the elderly. In older LTCF residents, anti-SARS-CoV-2 IgG antibody levels after BNT162b2 mRNA COVID-19 vaccination were higher in COVID-19 survivors than in uninfected individuals, especially after the first dose. It may suggest that elderly patients with a history of COVID-19 do not require a second dose of the BNT162b2 mRNA vaccine. The level of anti-SARS-CoV-2 IgG antibodies and its increase after vaccination with BNT162b2 mRNA COVID-19 did not correlate with the frailty and age of the study participants.

The datasets generated during the current study are available from the corresponding author on request.

## Supplementary Information

Below is the link to the electronic supplementary material.

Supplementary file1 (XLSX 27 kb)

## Data Availability

The datasets generated during the current study are available from the corresponding author on request.
